# The Impact of Anaesthesia Drugs and Techniques on Cancer Recurrence

**DOI:** 10.5152/TJAR.2022.22091

**Published:** 2022-10-01

**Authors:** Juan P. Cata, Ekin Güran

**Affiliations:** 1 Department of Anaesthesiology and Perioperative Medicine, University of Texas, MD Anderson Cancer Center, Houston, USA; 2 Anaesthesia and Surgical Oncology Research Group, Houston, USA; 3 Department of Anaesthesiology and Reanimation, University of Health Sciences, Ankara Oncology Training and Research Hospital, Ankara, Turkey

The burden of cancer incidence is increasing globally.^1^ In 2020, an estimated 18.1 million new cancer cases were diagnosed worldwide.^2^ If detected in early stages, most common cancers (i.e., lung, colorectal, liver, stomach, prostate, head and neck, and breast cancers) in adults can be cured or patients can sustain extended cancer-free survival after surgery alone or in combination with adjuvant therapies.

While surgery can be curative, there is evidence that it can also promote the growth and spread of micrometastasis or the “awakening” of dormant tumours.^3^ Surgical stress, inflammation, and immunosuppression in response to surgery have been implicated as the biological factors that can promote cancer progression in the perioperative period.^3^ However, it has been theorised that anaesthetics and different anaesthesia techniques administered during cancer surgery could also impact tumour growth and metastasis.^4^ Such theory has emerged from in vitro and in vivo experimental studies demonstrating that general and local anaesthetics could influence cancer cells’ behaviour or cells of the tumour microenvironment.^3-5^ To translate the results of those initial preclinical investigations, many retrospective studies and few randomised controlled trials were conducted over the last 2 decades.

One of the earliest studies tested the hypothesis that regional anaesthesia could reduce cancer progression by different mechanisms, including modulation of the sympathetic response, avoidance or reduction of general anaesthetics and opioids, and direct immunomodulatory effects of local anaesthetics.^6,7^ To date, the results of retrospective studies are conflicting. However, the body of evidence emerging from randomised controlled trials indicates that regional anaesthesia/analgesia has no clinical impact on cancer progression after mastectomies or major abdominal surgery for different cancers.^5,8^

It has also been speculated that lidocaine given intravenously during the duration of surgery and postoperatively could also influence cancer progression by enhancing the activity of immune cells such as natural killer cells, reducing the use of opioids, and acting directly on cancer cells in which it would induce apoptotic mechanisms or affect cell motility.^9^ These effects in vitro and in vivo; however, only 1 retrospective study has evaluated the association between the use of intravenous lidocaine on ovarian cancer progression. Interestingly, the authors reported that the intravenous use of lidocaine was associated with longer survival after ovarian cancer surgery.^10^ While the results from that study appear to be impactful, there is no evidence from randomised controlled trials supporting the short-term intraoperative use of lidocaine to improve oncological outcomes.

A group of investigators postulated and proposed the theory that volatile anaesthetics could also have a negative impact on oncological outcomes.^11^ On the other hand, they suggested that propofol would have the opposite effects. As a result, multiple retrospective studies and meta-analyses were conducted to determine the association between propofol-based intravenous anaesthesia and longer recurrence-free, progression-free, or overall survival after cancer surgery. The results of these investigations are conflicting. For example, Wigmore et al^12^ indicated that propofol-based intravenous anaesthesia improved the survival of patients undergoing surgery for abdominal metastatic or non-metastatic cancer. Contrarily, Makito et al^13^ using an extensive Japanese national registry, reported no clinically relevant differences in survival. Unfortunately, no data is available from well-designed randomised controlled trials evaluating the impact of propofol-based intravenous anaesthesia versus volatile-based anaesthesia on cancer recurrence.

The question that arises from the current evidence regarding the impact of any anaesthesia technique on cancer recurrence is why there are conflicting results from retrospective studies and less confusing answers from randomised controlled trials. An obvious answer is that retrospective clinical investigations suffer from significant biases, while randomised controlled trials account for most limitations. However, it can also be speculated that a major problem lies in the design of experimental studies investigating the effects of anaesthetics on cancer mechanisms related to tumour growth and metastasis. Such issues include large or repeated dosages of the tested drugs, cultured conditions not resembling the tumour microenvironment, and animals with immunological deficiencies to allow tumour engraftment. For instance, let us examine in vitro and in vivo studies demonstrating the anticancer effects of propofol. Hu et al^14^ indicated that propofol had in vitro antiproliferative effects in hepatocellular carcinoma cells. However, the range of doses of propofol (15-120 μM) used in that study was higher than the plasma concentrations of the drug to maintain general anaesthesia (11-22 μM). Therefore, we could conclude that drugs like propofol could have direct anticancer effects in doses associated with adverse effects (i.e., burst suppression or cardiovascular instability) in humans. On the other hand, 6-hour exposure to sevoflurane did not affect cell proliferation or metastasis in breast cancer cells.^15^

Alternatively, we could theorise that the anticancer effects of intravenous anaesthetics such as propofol could be mediated by a predominant impact on systemic immune effectors against cancer such as natural killer cells, as demonstrated in rodents. In contrast, volatile anaesthetics would impair the function of those cells.^16^ To address this question in the clinical setting, Hovaguimian et al^17^ conducted a randomised controlled trial in women with breast cancer. They measured natural killer cell activity and circulating tumour cell load before and after surgery under sevoflurane-based general anaesthesia or propofol-based general anaesthesia. The study demonstrated no differences in natural killer cell function or a correlation between function and circulating tumour cell load in both groups of patients.^18^ More recently, Oh et al^19^ randomised patients with colorectal cancer to sevoflurane-based general anaesthesia or propofol-based general anaesthesia and measured the fraction and apoptotic rate of circulating immunocytes by flow cytometry. No differences between groups were detected in the fraction of natural killer cells and T lymphocytes.

On the one hand, we could conclude from these studies that short-lived not-targeted perioperative interventions (mainly intraoperative or 1 or 2 days after surgery) do not influence immune makers, cancer cell behaviours, and more importantly, survival as previously implicated. On the other hand, we could hypothesise that pharmacologically targeted interventions administered in the perioperative period for a more extended period (weeks) could influence oncological outcomes. Clinical trials in humans support the evidence for this hypothesis.^19,20^ For instance, a multicentre, double-blind, placebo-controlled, randomised biomarker trial demonstrated that the administration of oral etodolac (400 mg) and propranolol (20 mg) 5 days before the day of the procedure and 5 days after breast cancer surgery reduced systemic inflammation and pro-metastatic biomarkers in the tumour and circulating immunocytes.^21,22^ Shaashua et al,^23^ who included patients undergoing colorectal cancer surgery, used the same strategy in a clinical investigation. Although the study enrolled 34 patients, the authors demonstrated that patients treated with etodolac and propranolol showed a favourable immune profile and a benefit in survival.

In conclusion, the use of intraoperative propofol or sevoflurane cannot explain differences in the mechanisms of cancer progression and different rates of survival between patients after cancer surgery. The use of regional anaesthesia should be indicated to provide adequate pain control during and after cancer surgery, but it does not modify patterns of cancer progression or survival. Perhaps, long-term (weeks) perioperative interventions focused on modulating inflammation (anti-inflammatory drugs) and reducing the stress response (beta-blocker) might confer survival benefits for patients undergoing cancer surgery.
